# French version validation of the psychotic symptom rating scales (PSYRATS) for outpatients with persistent psychotic symptoms

**DOI:** 10.1186/1471-244X-12-161

**Published:** 2012-09-28

**Authors:** Jerome Favrod, Shyhrete Rexhaj, Pascale Ferrari, Sabrina Bardy, Claude Hayoz, Stéphane Morandi, Charles Bonsack, Fabienne Giuliani

**Affiliations:** 1 La Source, Health Campus of the University of Applied Sciences of Western Switzerland, Lausanne, Switzerland; 2 Department of Psychiatry, Community Psychiatry Service, University Hospital Center of Lausanne, Lausanne, Switzerland; 3 Fondation HorizonSud, Marsens, Switzerland

**Keywords:** Hallucination, Delusion, Schizophrenia, PSYRATS, PANSS, Reliability, Validity

## Abstract

**Background:**

Most scales that assess the presence and severity of psychotic symptoms often measure a broad range of experiences and behaviours, something that restricts the detailed measurement of specific symptoms such as delusions or hallucinations. The Psychotic Symptom Rating Scales (PSYRATS) is a clinical assessment tool that focuses on the detailed measurement of these core symptoms. The goal of this study was to examine the psychometric properties of the French version of the PSYRATS.

**Methods:**

A sample of 103 outpatients suffering from schizophrenia or schizoaffective disorders and presenting persistent psychotic symptoms over the previous three months was assessed using the PSYRATS. Seventy-five sample participants were also assessed with the Positive And Negative Syndrome Scale (PANSS).

**Results:**

ICCs were superior to .90 for all items of the PSYRATS. Factor analysis replicated the factorial structure of the original version of the delusions scale. Similar to previous replications, the factor structure of the hallucinations scale was partially replicated. Convergent validity indicated that some specific PSYRATS items do not correlate with the PANSS delusions or hallucinations. The distress items of the PSYRATS are negatively correlated with the grandiosity scale of the PANSS.

**Conclusions:**

The results of this study are limited by the relatively small sample size as well as the selection of participants with persistent symptoms. The French version of the PSYRATS partially replicates previously published results. Differences in factor structure of the hallucinations scale might be explained by greater variability of its elements. The future development of the scale should take into account the presence of grandiosity in order to better capture details of the psychotic experience.

## Background

Psychosis is a complex disorder that expresses itself in a variety of different ways. The clinical assessment of the experience of psychosis is a challenge for health care professionals to communicate precisely amongst one another. It is also difficult to accurately measure the efficacy of treatments. Different scales have been developed to measure the presence and severity of symptoms of the illness. These tools, such as the Positive and Negative Syndrome Scale (PANSS) [1], the Scale for the Assessment of Positive Symptoms (SAPS) [2] or the Brief Psychiatric Rating Scale (BPRS) [3], measure a broad range of experiences and behaviours, something which restricts the detailed measurement of specific symptoms [4]. These scales have few items dedicated to the measurement of delusions or hallucinations, both of which are key symptoms of the psychotic experience. These symptoms are summarized as single broad scores without taking into account the multidimensional features of the experience. The various dimensions of the psychotic experience include the degree of conviction with which the delusional belief or beliefs about auditory hallucinations are held, the level of preoccupation it engenders, the degree of distress experienced as well as the behavioural responses used to cope with the experience [4]. These various dimensions may respond differentially to pharmacological [5-7] and psychological treatments [8-10].

Although there is some inconsistency in the literature about the chosen dimensions of delusions or hallucinations, assessment tools limited to a single item for key symptoms will miss large parts of the complex nature of such symptoms.

Haddock *et al.*[11] developed the Psychotic Symptom Rating Scales (PSYRATS) in order to improve the measurement of various dimensions of psychotic symptoms. The PSYRATS contains separate scales for auditory hallucinations and delusions. The PSYRATS auditory hallucination scale includes eleven items and the delusion scale includes six, all rated from zero to four. The psychometrics qualities of the PSYRATS indicate good internal consistency, sensitivity to changes, relationships with other measures of symptoms, inter-rater and retest reliability. The properties of the PSYRATS have been studied with various populations: hospitalized patients or day-treatment patients with first episode of schizophrenia or related disorders [12], recently relapsed patients with schizophrenia and schizoaffective disorders [4], hospitalized patients [13] and a mixed population of hospitalized patients and outpatients with schizophrenia and schizoaffective disorders [11], and patients with mental retardation [14]. The auditory hallucination scale has also been validated in Spanish [15].

In the initial study, the factor analysis yielded three independent factors for the auditory hallucination scale: emotional characteristics, physical characteristics and cognitive interpretation [11]. Two factors were reported in relation to the delusion scale, designated as emotional characteristics and cognitive interpretation (Haddock *et al.* 1999). The PSYRATS has been used as outcome measures in a number of studies aimed at evaluating the effectiveness of psychological interventions for psychosis [16-19]. It has also been used to quantify the impact of psychotic symptoms on the cognitive process [20,21]. However, the PSYRATS factor subscales have not been consistently used. This may be due to the reported lack of consistency in replications of the first factor solution [4,11,13,12]. Whilst the PSYRATS scales provide a total cumulative score for each symptom, the multidimensional nature of the symptoms suggests that the total score should not be presented alone. This has driven some researchers to report their use of PSYRATS on the basis of single items [22]. The use of single items has also been recommended to assess specific changes during therapy [4]. The aim of this study was to examine the psychometric properties of the French version of the PSYRATS in a sample of patients suffering from schizophrenia spectrum disorders with persistent psychotic symptoms. The two scales of the PSYRATS were evaluated for inter-rater reliability and inter-relationships of the items. In addition, construct and concurrent validity were examined.

## Methods

### Recruitment and ethical approval

Participants were recruited from three studies on persistent psychotic symptoms [9,23-25]. To be included in the studies, participants had to meet the criteria for ICD-10 schizophrenia or schizoaffective disorders. Other inclusion criteria for these studies were the presence of delusions or hallucinations greater than 2 on the PANSS item P1 (delusion) or P3 (hallucination). Participants needed to be experiencing these symptoms for the three months prior to the study and with no change in antipsychotic medication during the two months before inclusion. Participants were recruited from among the patients followed by the Rehabilitation unit of the Community Psychiatry Service of the Department of Psychiatry at the University Hospital of Lausanne and from the Foundation HorizonSud in Marsens, both in Switzerland. The Rehabilitation unit of the Community Psychiatry Service is a comprehensive, recovery-oriented outpatient program that provides a variety of services organized around clinical case management.

HorizonSud is a foundation that provides a sheltered workshop, transitional employment, and sheltered accommodation in a range from nursing home accommodation to independent apartments. The three studies from which the sample was drawn received approval by the ethics committee at the University of Lausanne and all participants signed an informed consent form.

### Participants

One hundred and three participants took part in the study. Eligibility depended on the presence of persistent delusions or auditory hallucinations during the previous three months before consent was obtained. 41 (40%) were female and 62 (60%) male. Diagnoses were extracted from current clinical records and confirmed by experienced clinicians; schizophrenia (90%) or schizoaffective disorder (10%). The mean age was 36.9 years (SD = 9.8). Both auditory hallucinations and delusions were reported by 48 participants. Ninety-four participants reported having delusions, among them 46 reported delusions without auditory verbal hallucinations. Fifty-five participants reported having auditory verbal hallucinations, among them 9 without delusion. Eighty-one participants have never been married, 10 were currently married, 2 separated and 10 divorced. Three were working on the competitive job market, 22 were unemployed and 69 were working in sheltered workshops. The others were working as volunteers, housewives or students. All participants spoke French fluently. Seventy-five participants were interviewed using the items of the positive symptoms scale of the PANSS.

### Instruments

Patients were assessed using the following instruments:

• Psychotic Symptom Rating Scales [11]. The PSYRATS is a 17-item, five-point scale (0–4), multidimensional measure of delusions and auditory hallucinations. Symptoms from the previous week were rated. Two scores were obtained: Auditory Hallucinations Scale (11 items) and Delusions Scale (6 items). The items for auditory hallucinations are: frequency, duration, location, loudness, beliefs about origin, negative content, intensity of negative content, amount of distress, intensity of distress, disruption of life and control. The items for delusions are: amount of preoccupation, duration of preoccupation, conviction, amount of distress, intensity of distress and disruption of life. The original English version of the PSYRATS was independently translated by two mother tongue French-speaking members of our workgroup and compared until full agreement was found. The translation was authorized by the main author of the original version.

• Positive and Negative Syndrome Scale (PANSS) [1,26,27]. The PANSS is a 30-item, seven point (1–7) rating instrument used for the assessment of phenomena associated with schizophrenia. Symptoms from the previous two weeks were rated. Seventy-five participants included in two studies were assessed using the items of the positive symptoms scale, and the anxiety (G2) and depression (G6) items of the general psychopathology scale.

For all patients, symptom rating assessments were performed by clinicians trained to reliably administer these measures. Regular random tests of inter-rater reliability were conducted between the raters by independent ratings of 2 raters for 15 assessments.

### Statistical analysis

All analyses were conducted using IBM SPSS Statistics version 20. The factor structure of the scales was evaluated by principal component factor analysis with a single varimax rotation with Kaiser Normalization. Inter-rater reliability was assessed by intraclass correlations (ICCs). Significance test results are quoted as two-tailed probabilities. Associations between PSYRATS and PANSS items were examined using Spearman rank-based correlations.

## Results

The fifty-five participants with auditory hallucinations had a mean score of 26.5 (SD = 7.6; range = 8–38) on the auditory hallucination scale. The 94 participants with delusions had a mean score of 15.1 (SD = 3.6; range: 3–24) on the delusions scale. High scores are indicative of more severe symptoms.

### Inter-rater reliability

Intraclass correlations for the positive symptom items of the PANSS were good to excellent. ICC were: 0.87 for delusion, 0.74 for conceptual disorganization, 0.95 for hallucination, 0.82 for hyperactivity, 0.95 for grandiosity, 0.87 for suspiciousness/persecution and 0.73 for hostility. For the delusions scale of the PSYRATS, ICCs were excellent, between 0.92 to 1.00. For the hallucinations scale the ICCs were also excellent, between 0.90 to 1.00. For both scales, the lowest ICC was obtained for the disruption to life item.

### Inter-relationships items

For the delusions scale, four of the 15 correlations were significant at the .01 level. Amount of preoccupation was correlated with the duration of preoccupation (0.47) and disruption to life (0.45); duration of preoccupation with disruption to life (0.42); amount of distress with intensity of distress (0.57). For the hallucinations scale, fifteen of the 55 correlations were significant at the .01 level, as follows. Frequency correlated with intensity of distress (0.40) and disruption to life (0.39); location with origin of voice (0.45); loudness with disruption to life (0.37); origin of voice with degree of negative content (0.54), intensity of distress (0.37) and controllability (0.43); amount of negative content with degree of negative content (0.56), amount of distress (0.75), and intensity of distress (0.49); degree of negative content with amount of distress (0.67), intensity of distress (0.53), disruption to life (0.37) and controllability (0.33); amount of distress with intensity of distress (0.77).

### PSYRATS hallucination factor analysis

The factor structure of the auditory hallucinations was explored using a principal component factor analysis with a single varimax rotation. Only the participants who reported auditory verbal hallucinations were included in the factor analysis of the PSYRATS hallucinations scale items. A four-factor solution appears as an optimal representation of the data structure (Kaiser criterion). This solution explained 72% of the total variance. These four factors could be identified as: an emotional characteristics factor (factor 1), a cognitive interpretation factor (factor 2), a disruption factor (factor 3) and a physical characteristics factor (factor 4). (See Table 1).

**Table 1:** **PSYRATS Hallucination scale factoranalysis**

	**1**	**2**	**3**	**4**
Frequency				0.93
Duration				0.44
Location		0.78		
Loudness			0.78	
Origin of voice		0.81		
Amount of negative content	0.86			
Degree of negative content	0.76			
Amount of distress	0.93			
Intensity of distress	0.79			
Disruption to life			0.72	
Controllability		0.54		
Eigenvalues	4.1	1.6	1.2	1.0
Cumulative percentage of variance	37.6	52.0	62.5	71.9

### PSYRATS delusion factor analysis

Factor analysis of the delusion scale items using a principal components factor analysis with a single varimax rotation identified two factors. Only those participants who reported delusions were included in the factor analysis of the PSYRATS delusions scale items. A two factor solution appears as an optimal representation of the data structure (Kaiser criterion). This solution explained 63 % of the total variance. The items tend to constitute a cognitive interpretation factor (factor 1) and a distress factor (factor 2). (See Table 2).

**Table 2:** **PSYRATS Delusion scale factoranalysis**

	**1**	**2**
Amount of preoccupation	0.76	
Duration of preoccupation	0.74	
Conviction	0.41	
Amount of distress		0.74
Intensity of distress		0.73
Disruption to life	0.65	
Eigenvalues	2.3	1.5
Cumulative percentage of variance	38.5	63.3

### Convergent validity: relationships with other measures of symptoms

Spearman rank correlations coefficients were calculated between the PSYRATS auditory hallucinations and items of the PANSS. The frequency of voice, origin of voice, degree of negative content items and the total score of the PSYRATS hallucinations scale were correlated at the .01 level with the P3 hallucination item of the PANSS. Duration, amount of distress, and intensity of distress items were correlated at the .05 level with the P3 hallucination item of the PANSS (see Table 3). The disruption to life item of the PSYRATS hallucination scale was significantly correlated with G6 depression (r = 0.35, 2-tailed p = .03) and G2 anxiety (r = 0.50, 2-tailed p = .001) items. The amount of preoccupation and the conviction items and the total score of the PSYRATS delusions scale were correlated at the .01 level with the P1 delusion item of the PANSS. The duration of preoccupation item of the PSYRATS delusions scale was correlated at the .05 level with the P1 delusion item of the PANSS. The amounts of distress, intensity of distress and disruption to life items and the total score of the PSYRATS delusions scale were also correlated with the G6 depression items of the PANSS (see Table 4). The G2 Anxiety item of the PANSS was correlated with the disruption to life item and the total score of the PSYRATS delusions scale. The P5 grandiosity item of the PANSS was significantly negatively correlated with the amount of distress and intensity of distress items of the PSYRATS delusions scale. The P6 suspiciousness/persecution item of the PANSS was correlated with the amount of preoccupation, duration of preoccupation, disruption to life and total score of the PSYRATS delusions scale.

**Table 3:** **Correlations between items ofthe PSYRATS hallucination scaleand PANSS P3 Hallucinationitem**

	**PANSS P3 Hallucination (N** **= 39)**
Frequency	0.42**
Duration	0.33*
Location	0.07
Loudness	0.30
Origin of voice	0.46**
Amount of negative content	0.28
Degree of negative content	0.55**
Amount of distress	0.33*
Intensity of distress	0.34*
Disruption to life	0.26
Controllability	0.31
Total	0.58**

*2-tailed p < 0.05.

**2-tailed p < 0.01.

**Table 4:** **Correlations between items ofthe PSYRATS delusion scaleand PANSS items**

	**PANSS P1 Delusion**	**PANSS G6 Depression**	**PANSS G2 Anxiety**	**PANSS P5 Grandiosity**	**PANSS P6 Suspiciousness/persecution**
	**(N = 72)**	**(N = 72)**	**(N = 72)**	**(N = 72)**	**(N = 72)**
Amount of preoccupation	0.41**	0.08	0.11	0.16	0.40**
Duration of preoccupation	0.27*	0.13	0.30*	0.16	0.31*
Conviction	0.47**	0.02	0.15	0.19	0.15
Amount of distress	0.02	0.38**	-0.05	-0.25*	0.17
Intensity of distress	0.13	0.43**	0.17	-0.24*	0.14
Disruption to life	0.14	0.31**	0.42**	0.07	0.35**
Total PSYRATS delusion	0.37**	0.45**	0.29*	-0.03	0.43**

*2-tailed p < 0.05.

**2-tailed p < 0.01.

## Discussion

The current study presents the factor structure and the relationship between items of the PSYRATS and items of the PANSS with a population of French-speaking outpatients having persistent psychotic symptoms. The participants showed a total score close to that of recently relapsed patients [4]. The factor structure of the delusions scale of the PSYRATS is the same as in the original study [11], the German version study [13] and the first episode psychosis study [12]. The factor structure of the hallucinations scale shows a four- factor structure. Three of them—emotional characteristics factor, cognitive interpretation factor and physical characteristics factor—appear as in previously published studies [4,11,13]. The factor analysis for our study encountered the same difficulties as previous studies did when replicating the factorial structure of the original version of the hallucinations scale. As in previously published studies, an emotional factor appears clearly. [4,11-13]. The frequency and duration items of auditory hallucination load on a physical factor as in previous studies. The belief about voice items load on a cognitive interpretation factor as in previous studies. Also, similar to previously published studies, the location factor loads on a cognitive factor [4,12,13]. The disruption to life item and the loudness item load on a disruption factor as in the Steel *et al.* study [4]. The controllability item load on a cognitive factor close to the Drake *et al.* study [12]. As suggested by Steel *et al.*[4], we probably lack a clear understanding of the dimensions of hallucinatory experience. Moreover, the different evaluations of the PSYRATS have been carried out with dissimilar participant populations with psychotic symptoms. The difficulty to replicate the factor structure of the auditory hallucinations scale of the PSYRATS with different populations may be explained by a differential response between hallucination and delusion to psychiatric treatment. For example, Schneider *et al.*[7] have shown with patients suffering from schizophrenia and auditory verbal hallucinations that in the longitudinal course, a general symptomatic decrease became apparent only for auditory hallucinations but not for delusions. Loudness of the voice and distress associated with auditory hallucination decreased early compared to other aspects of hallucination that took more time to fade. Chang *et al.*[28] have described that cluster structure stabilizes after 6 months of antipsychotic treatment, and the changes in physical characteristics may not be the sole sign of clinical improvement. The difficulty in replicating the factor structure may also be explained by the heterogeneous nature and complex nature of auditory hallucinations [29].

For the concurrent validity, the total score of the PSYRATS hallucinations significantly correlate with the P3 item (hallucination) of the PANSS. The total score of the PSYRATS delusions significantly correlate with the P1 and P6 of the PANSS. Some individual items of the PSYRATS hallucinations or delusions scales do not respectively correlate with the P3 Hallucination item and P1 Delusion item of the PANSS, suggesting that this last item reflects selected aspects of psychotic symptomatology as already demonstrated by Steel *et al.*[4] on a larger sample. In the current study, the P1 item of the PANSS fails to assess the amount of distress and the intensity of the distress associated with delusions. These items are more related with the G6 depression item of the PANSS. Inversely, the PSYRATS delusions scale does not appear to take into account the elements associated with delusions of grandiosity as measured with the P5 item of the PANSS. The amount of distress and intensity of distress items are negatively associated with grandiosity. A more detailed assessment of emotional and behavioural reactions to grandiosity would be worthwhile, in order to measure the effects of new psychological interventions in this area. Grandiosity delusions are associated with poor medication compliance [30]. The same comment could apply to benevolent voices and the auditory hallucination scale of the PSYRATS, which does not take into account the particular emotional and behavioural reactions to that kind of voice [31].

The present study does not replicate the Steel *et al.* failure of the P3 item of the PANSS to assess the negative content and distress associated with hallucinations [4]. This might be explained by a population difference and the fact that our sample of participants with hallucinations who were also assessed with the PANSS was smaller.

The disruption to life items of the PSYRATS scales need refinement. The anchor point of the items have varied between studies [4,11] and there is a difference in precision of the description of the clinical observations in comparison to the other items which are described more precisely. In the present study, the lower inter-rater ICCs were obtained for these two items. This may lead to differences between the different factor analyses published until yet.

A first limitation of the current study was the relatively small sample of participants, something which may have prevented the replication of previously published results. The fact that participants were administered a French version of the PANSS may also have contributed to some of the unique findings of this study. We selected participants with persistent psychotic symptoms, an often neglected population that is not easy to reach. Beyond the specific interest for this subpopulation, this was also a limitation of the study since results may not be replicated in a normally distributed population of persons suffering from schizophrenia. For the external validity, the smaller number of participants with persistent hallucinations compared to participants with persistent delusions might also have been a limitation of the present study.

## Conclusion

In conclusion, the French version of the PSYRATS partially replicates previous evaluations of the scale in English or German. As in previous studies the factor structure of the auditory hallucination scale appears less stable. The longitudinal course of auditory hallucinations need further research to better understand how the factor structure of the scale varies across time. The disruption to life items of both scales should be more detailed to better tape the specific behavioural consequences of psychotic symptoms. The future development of the scale should take into account the emotional and behavioural consequences of delusions associated with grandiosity and benevolent voices in order to capture better important parts of the psychotic experience.

## Competing interests

The authors declare that they have no competing interests.

## Authors' contributions

JF, PF, SB, and CH contributed to the conception and design of the study. SR and SB contributed to the acquisition of the data. JF, SR and FG contributed to data analysis and interpretation of the data. JF and SR drafted the manuscript. PF, SB, CH, SM and CB were involved in the critical revision of the manuscript. All authors have given final approval of the version to be published.

## Pre-publication history

The pre-publication history for this paper can be accessed here:

http://www.biomedcentral.com/1471-244X/12/161/prepub
